# Hepatic transcriptome perturbations in dairy cows fed different forage resources

**DOI:** 10.1186/s12864-020-07332-0

**Published:** 2021-01-07

**Authors:** S. T. Gao, Lu Ma, Y. D. Zhang, J. Q. Wang, J. J. Loor, D. P. Bu

**Affiliations:** 1 State Key Laboratory of Animal Nutrition, Institute of Animal Science, Chinese Academy of Agricultural Sciences, No. 2 Yuanmingyuan West Road, Beijing, 100193 China; 2 Department of Animal Sciences and Division of Nutritional Sciences, University of Illinois, Urbana, IL USA

**Keywords:** Liver transcriptome, Forage resources, RNAseq, Corn Stover

## Abstract

**Background:**

Forage plays critical roles in milk performance of dairy. However, domestic high-quality forage such as alfalfa hay is far from being sufficient in China. Thus, more than 1 million tons of alfalfa hay were imported in China annually in recent years. At the same time, more than 10 million tons of corn stover are generated annually in China. Thus, taking full advantage of corn stover to meet the demand of forage and reduce dependence on imported alfalfa hay has been a strategic policy for the Chinese dairy industry. Changes in liver metabolism under different forage resources are not well known. Thus, the objective of the present study was to investigate the effect of different forage resources on liver metabolism using RNAseq and bioinformatics analyses.

**Results:**

The results of this study showed that the cows fed a diet with corn stover (CS) as the main forage had lower milk yield, DMI, milk protein content and yield, milk fat yield, and lactose yield than cows fed a mixed forage (MF) diet (*P* <  0.01). KEGG analysis for differently expressed genes (DEG) in liver (81 up-regulated and 423 down-DEG, Padj ≤0.05) showed that pathways associated with glycan biosynthesis and metabolism and amino acid metabolism was inhibited by the CS diet. In addition, results from DAVID and ClueGO indicated that biological processes related to cell-cell adhesion, multicellular organism growth, and amino acid and protein metabolism also were downregulated by feeding CS. Co-expression network analysis indicated that *FAM210A*, *SLC26A6*, *FBXW5*, *EIF6*, *ZSCAN10*, *FPGS*, and *ARMCX2* played critical roles in the network. Bioinformatics analysis showed that genes within the co-expression network were enriched to “pyruvate metabolic process”, “complement activation, classical pathway”, and “retrograde transport, endosome to Golgi”.

**Conclusions:**

Results of the present study indicated that feeding a low-quality forage diet inhibits important biological functions of the liver at least in part due to a reduction in DMI. In addition, the results of the present study provide an insight into the metabolic response in the liver to different-quality forage resources. As such, the data can help develop favorable strategies to improve the utilization of corn stover in China.

**Supplementary Information:**

The online version contains supplementary material available at 10.1186/s12864-020-07332-0.

## Background

Forage is the largest component of the diet of lactating cows and could affect dry matter intake (DMI) [1] and consequently milk performance. Nutrient content such as crude protein (CP), neutral detergent fiber (NDF), and non-fibrous carbohydrate (NFC) of different forages differ greatly. For instance, alfalfa hay, a well-known high quality forage, contains higher CP, rumen degradable protein (RDP), and rumen undegradable protein (RUP) content than corn stover and Chinese wild rye grass [2]. Increasing RUP by 1% can improve milk production by 1 kg [3]. In addition, cows fed high proportions of alfalfa hay have higher milk protein production by increasing microbial protein yield, which may be attributed to the increased supply of rumen-available energy [2].

While high-quality forage such as alfalfa hay is still a bottleneck for the development of the dairy industry in China, it has become one of the largest dairy producers in the world [4]. In 2019, more than 1.2 million tons of alfalfa hay were imported to cover the shortage of high-quality forage in China [5]. At the same time, it is estimated that more than 10 million tons of corn stover are generated annually in China [6]. Thus, taking full advantage of crop residues such as corn stover to meet the demand of forage and reduce dependence on imported alfalfa hay has been a strategic policy for the Chinese dairy industry [2].

In the last 10 years, a large number of studies to evaluate the nutritive value of corn stover or Chinese wild rye grass have been conducted [2, 4, 7, 8]. For instance, Zhu et al. (2013) investigated the effect of different forage sources on lactation performance, microbial protein (MCP) synthesis, and N utilization efficiency in early lactation dairy cows [2]. Through studying metabolites from four biofluids (rumen fluid, milk, serum, and urine), Sun et al. (2015) elucidated the metabolic mechanisms of milk production affected by forage quality [4]. Zhang et al. (2014) evaluated the effects of diets with three different quality forage sources (alfalfa hay, *L. chinensis* and cornstalk) on the rumen microbiota of dairy cows [7].

In ruminants, liver contributes to more than 80% of the glucose produced via gluconeogenesis [9, 10]. In addition, liver is a critical hub for numerous physiological processes including lipid metabolism, amino acid metabolism, detoxification, and immune defense [11, 12]. Overall function and metabolism of the liver are sensitive to the plane of nutrition of the cows. For instance, Shahzad et al. (2014) demonstrate that the liver of cows fed a diet to meet 80% of estimated requirements had greater lipid and amino acid catabolic capacity and a more pronounced cellular inflammatory and endoplasmic reticulum stress response, while the liver of cows fed to meet or exceed requirements had a larger cell proliferation and cell-to-cell communication and greater activation of pathways/functions related to triacylglycerol synthesis [13]. Previous studies have been mainly focused on the effect of different forage resources on lactation performance and rumen fermentation, but simultaneous changes in liver metabolism under different forage resources are not well known. Thus, the objective of the present study was to investigate the effect of different forage resources on liver metabolism using RNAseq and bioinformatics analyses.

## Results

### Milk performance of cows fed different forage resources

As shown in Table 1, milk yield (30.5 vs. 23.1 kg/d, *P* <  0.01) and efficiency (1.47 vs. 1.32%, *P* <  0.01) was lower with CS than MF. In addition, DMI (21.4 vs. 17.4 kg/d, *P* <  0.01), milk protein content and yield (3.66 vs. 3.32%, *P* <  0.01;1.11 vs. 0.77 kg/d, *P* <  0.01), milk fat yield (1.34 vs. 1.02 kg/d, *P* <  0.01), and lactose yield (1.47 vs. 1.13 kg/d, *P* <  0.01) were all decreased by CS compared with MF.

**Table 1:** Milk yield and composition of lactating cows fed diets based on different forage sources

Items	Treatments		SEM	*P*-value
	MF	CS		
DMI, kg/d	21.4	17.4	0.14	< 0.01
Milk yield, kg/d	30.5	23.1	0.90	< 0.01
Protein, %	3.66	3.32	0.07	< 0.01
Protein yield, kg/d	1.11	0.77	0.03	< 0.01
Fat, %	4.46	4.38	0.13	0.65
Fat yield, kg/d	1.34	1.02	0.03	< 0.01
Lactose, %	4.86	4.80	0.03	0.09
Lactose yield, kg/d	1.47	1.13	0.04	< 0.01
Efficiency^a^, %	1.47	1.32	0.04	< 0.01

^a^Efficiency = Milk yield/DMI

### Differently expressed genes (DEG) and functional analysis

A total of 8582 unigenes were detected in the liver of dairy cows and 504 DEG (81 up- regulated and 423 down-DEG, Padj ≤0.05) were identified between cows consumed CS and MF (Additional File 1). Functional analysis for the DEG was performed using DIA, DAVID, and ClueGO.

**Fig. 1:**
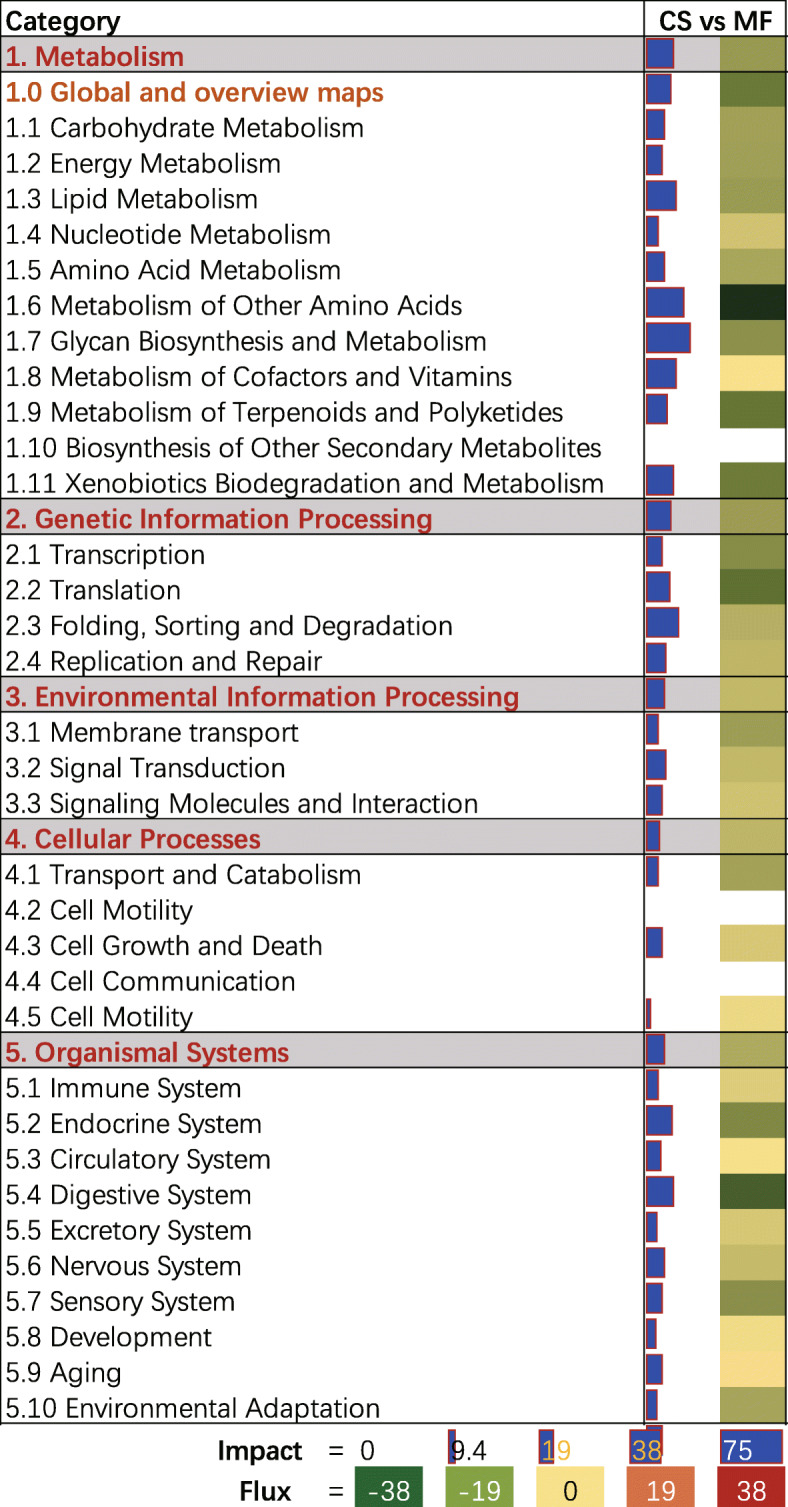
Summary of the main categories and sub-categories of KEGG pathways as results of the transcriptomic effect on liver tissue of corn stover (CS) compared to mixed forage (MF) as analyzed by the Dynamic Impact Approach. On the right are the bar denoting the overall impact (in blue) and the shade denoting the effect on the pathway (from green – inhibited – to red – activated). Darker the color larger the activation (if red) or inhibition (if green) of the pathway

**Fig. 2:**
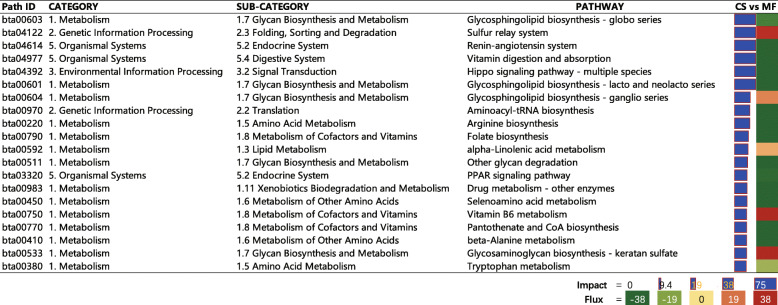
The 20 most impacted pathways in liver tissue of corn stover (CS) compared to mixed forage (MF) uncovered by the Dynamic Impact Approach. On the right are the bar denoting the overall impact (in blue) and the shade denoting the effect on the pathway (from green – inhibited – to red – activated). Darker the color larger the activation (if red) or inhibition (if green) of the pathway

The whole DIA output is available in Additional File 1. As shown in Fig. 1 where the perturbation in CS cows vs. MF cows on the main categories of the KEGG pathways in liver is summarized, all categories and subcategories were inhibited to different extents. For instance, “Metabolism” followed by “Genetic Information Processing” and “Environmental Information Process” were the most impacted. Within the most impacted category of “Metabolism” (Fig. 1), the subcategory “Glycan Biosynthesis and Metabolism” was the most impacted and was overall inhibited. Among the top 20 impacted pathways in liver tissue of CS compared with MF cows uncovered by the DIA, most of the pathways were inhibited (Fig. 2). Few pathways such as “Sulfur relay system”, “Vitamin B6 metabolism”, and “Glycosaminoglycan biosynthesis-keratan sulfate” were highly activated, and “Glycosaminoglycan biosynthesis-ganglio series” and “alpha-Linolenic acid metabolism” were modestly activated. Furthermore, among the top 20 most impacted pathways, approximately 25% were related to “Glycan Biosynthesis and Metabolism” with the pathway of “Glycosphingolipid biosynthesis – globo series” being the most impacted (Fig. 2).

**Fig. 3:**
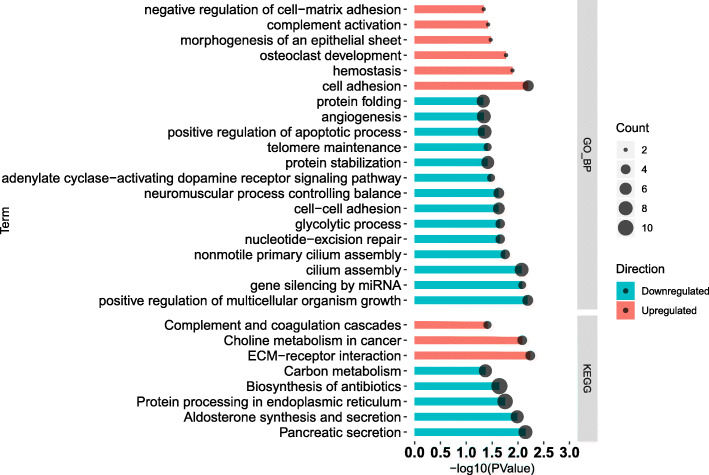
Significantly enriched Gene Ontology Biological Process and KEGG pathways revealed by DAVID analysis of the transcripts up- (in red shade in the figure) or down- (in blue shade in the figure) regulated in liver tissue of corn stover (CS) compared to mixed forage (MF) cows. In vertical axis is the terms, in horizonal axis is the transformed FDR (−log10PValue)

Results of DAVID analysis are shown in Fig. 3 where KEGG and GO Biological Process (GO_BP) analysis were conducted. The GO_BP analysis revealed that 14 and 6 different terms (*P* ≤ 0.05) were enriched by downregulated DEG and upregulated DEG respectively. For the KEGG analysis, there were 8 terms enriched among DEG in total, with 5 terms enriched with downregulated DEG (*P* ≤ 0.05).

**Fig. 4:**
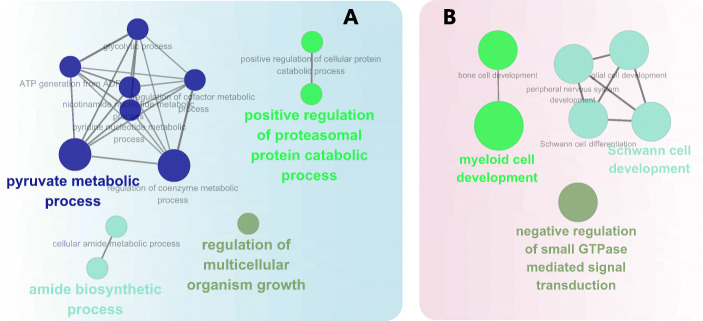
Functional annotation of DEGs using ClueGO. **a**: Enriched by downregulated DEGs; **b**: Enriched by upregulated DEGs. Each node is a Gene Ontology (GO) Biological Process term. The size of the nodes reflects the statistical significance of each term. Larger the node size, smaller the *P*-value. Different node colors represent different functional groups. The name of each group is given by the most significant term of the group. The nodes are grouped by similarity of their associated genes

The GO_BP analysis was also performed using ClueGO (Fig. 4). The results show that downregulated DEG were enriched to “pyruvate metabolic process”, “positive regulation of proteasomal protein catabolic”, “amide biosynthetic process”, and “regulation of multicellular organism growth”, while the upregulated DEG were enriched to “myeloid cell development”, “Schwann cell development”, and “negative regulation of small GTPase mediated signal transduction” (*P* ≤ 0.05).

### Co-expression Network and Functional analysis.

**Fig. 5:**
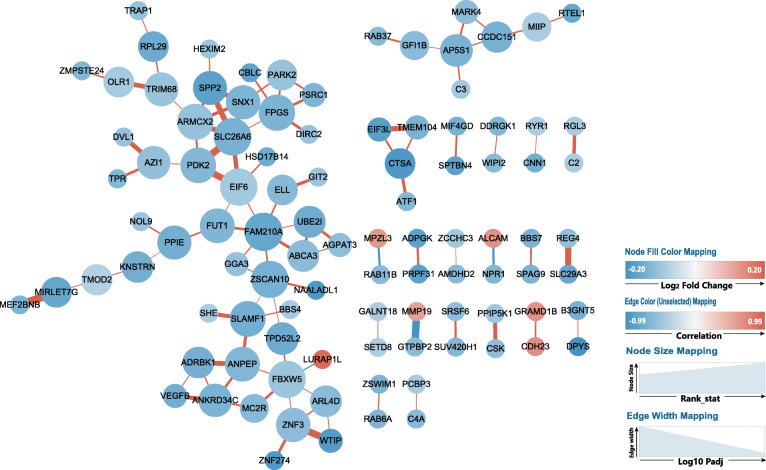
Co-expression networks constructed using differently expressed genes (DEG) with absolute correlation ≥0.9 and adjusted *p*-value ≤0.01 by Cytoscape. The color of the nodes represents the fold change of the gene expressed in mixed forage (MF) compared to corn stover (CS). Upregulated genes are in red color, downregulated genes in blue color. Deeper the color, higher the fold changes. The size of the nodes represents the combined ranking of the degree and betweenness of the nodes (genes) in the network. Larger the size, higher the ranking. Color of the edges represent the correlation between the genes. Positive correlation is in red, negative correlation is in blue. The width of the edge represents significance of the correlation between the two genes. Larger the width, smaller the Padj

**Fig. 6:**
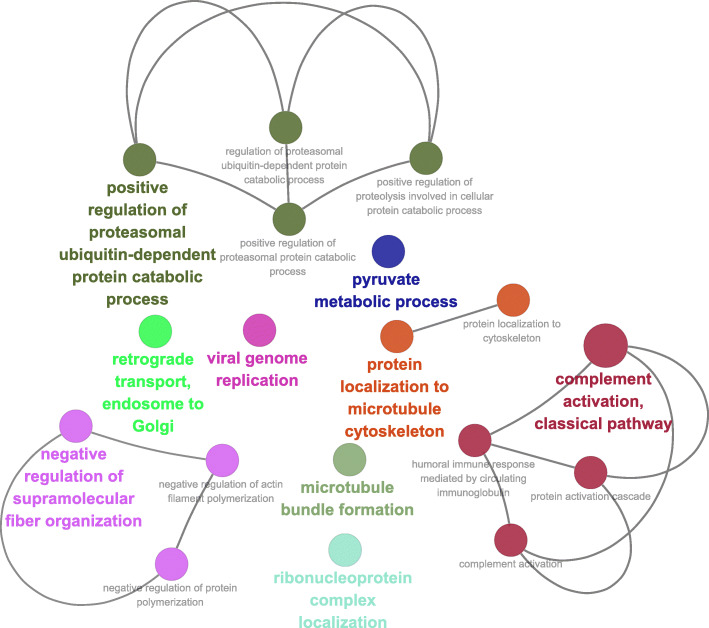
Gene Ontology Biological Process (GO_BP) annotation for the whole co-expression network. The size of the nodes reflects the statistical significance of each term. Larger the node size, smaller the *P*-value. Different node colors represent different functional groups. The name of each group is given by the most significant term of the group. The nodes are grouped by similarity of their associated genes

Co-expression network analysis provides insights into the patterns of transcriptome organization and can reveal common biological functions among network genes [14]. The co-expression network analysis of this study was conducted using DEG with correlation > 0.9 and Padj < 0.01 (Additional file 4). The entire co-expression network is shown in Fig. 5, and the annotation information of the genes is available in Fig. 6. As shown in Fig. 5, the co-expression network revealed 7 genes (*FAM210A*, *SLC26A6*, *FBXW5*, *EIF6*, *ZSCAN10*, *FPGS*, *ARMCX2*) with higher degree and betweenness centrality (ranking in top 7, Additional file 5) than others, indicating a more critical role played by them in the network.

Annotation information analysis for the genes within the co-expression network was performed using ClueGO and is shown in Fig. 6. Genes within the whole network were significantly enriched in “complement activation, classical pathway”, “retrograde transport, endosome to Golgi”, “positive regulation of proteasomal ubiquitin-dependent protein catabolic process”, “microtubule bundle formation”, “negative regulation of supramolecular fiber organization”, “viral genome replication”, “protein localization to microtubule cytoskeleton”, “ribonucleoprotein complex localization”, and “pyruvate metabolic process” (Fig. 6).

## Discussion

Liver plays a central role in supporting the anabolic capacity of the mammary gland. Net hepatic glucose production (3.1 kg/d) of mid-to-late lactating cows is able to meet glucose required for milk lactose synthesis and maintenance [15, 16]. In addition, liver plays dominant roles in determining the ultimate quantity and pattern of metabolites available for milk synthesis [16]. Metabolic function and, thus, energy metabolism of liver responds to a variety of environmental stimuli including fasting or level of feed intake [17], diet composition and productive (physiological) state [15]. Although a number of studies have been conducted to assess effects of low-quality forage resources on lactation performance and rumen fermentation [2, 4, 7, 8], there are limited data on the response by important organs such as the liver. Thus, we used transcriptomics and bioinformatics in an effort to better capture genome-wide transcriptional responses of dairy liver to feeding low-quality forage (CS) versus high-quality forage (MF).

### Feeding CS reduces Milk performance

Consistent with a previous study where milk yield of cows fed more alfalfa than those fed corn stover (*P* = 0.07) decreased [2], in this study milk yield was lower with CS than MF (Table 1). In addition, milk protein content and yield, milk fat yield, and lactose yield were all decreased by CS compared with MF (Table 1). Clearly, a large portion of the decreased milk performance in this study was mainly attributed to the lower DMI of CS compared with MF cows [18]. The study of Zhu et al. (2013) showed that corn stover compared with alfalfa led to lower OM degradability in the rumen (53.2 vs. 47.8%, *P* = 0.01) [2], suggesting longer retention time of undegraded fiber. Thus, the lower DMI of CS vs. MF cows in this study was likely caused by excess bulk in the rumen.

### Pathways in liver were extensively inhibited in CS cows vs. MF cows

In this study, all categories and subcategories of the KEGG pathways in liver were overall inhibited to different extents in CS vs. MF cows (Fig. 1). Furthermore, among the top 20 impacted pathways, in liver tissue of CS compared with MF cows uncovered by the DIA, most of the pathways were inhibited (Fig. 2). Data for inhibited pathways indicated an overall downregulated metabolism in liver of CS compared with MF cows, which agrees with results of Sun et al. (2015) in which ruminal fluid and serum metabolite concentrations decreased with a low-forage compared with high-forage diet [4]. Thus, together the data imply a decreased overall metabolism level when low-quality forage is fed.

### Low-quality forage inhibited glycan biosynthesis and metabolism

As shown in Fig. 1, the subcategory “Glycan Biosynthesis and Metabolism” was the most impacted and was overall inhibited. Furthermore, among the top 20 most impacted pathways, approximately 25% were related to “Glycan Biosynthesis and Metabolism” with the pathway of “Glycosphingolipid biosynthesis – globo series” being the most impacted (Fig. 2). Glycans are simple or complex polymers composed of monosaccharides [19], and mediate a wide variety of biological processes including cell growth and differentiation, cell−cell communication, immune response, pathogen interaction, and intracellular signaling events [20]. At a molecular level, glycans are often the first points of contact between cells, and they function by facilitating a variety of interactions both in cis (on the same cell) and in trans (on different cells) [21]. Thus, the high perturbation of glycan biosynthesis and metabolism in this study suggests a potential effect of low-quality forage on hepatocyte communication or growth and differentiation, which was also validated by the results of DAVID and ClueGO where the biological process of “cell-cell adhesion” and “positive regulation of multicellular organism growth” were significantly enriched among the downregulated DEG (Fig. 3 and Fig. 4).

Among the top 20 impacted pathways, “Glycosphingolipid biosynthesis – globo series” and “Glycosphingolipid biosynthesis – lacto and neolacto series” were highly inhibited in CS vs. MF cows (Fig. 2). In addition, “Glycosphingolipid biosynthesis – ganglio series” was also highly impacted, but the change in direction of the DEG involved in the pathway was not consistent, which is embodied in the modest direction of the impact (Fig. 2). However, it was evident that glycosphingolipid biosynthesis metabolism was overall inhibited by CS vs. MF in this study. Glycosphingolipids (GSLs) comprise a heterogeneous group of membrane lipids formed by a ceramide backbone covalently linked to a glycan moiety [22], and are classified based on their carbohydrate structure into six major series in vertebrates including gangliosides, lacto-, neolacto-, muco-, isoglobo-, and globo-series GSL [23]. D’Angelo et al. (2013) compiled published data indicating that GSL could modulate various aspects of the biology of the cell including apoptosis, cell proliferation, endocytosis, intracellular transport, cell migration and senescence, and inflammation [22]. Zhang et al. (2004) concluded that specific changes in composition and metabolism of GSL occur during cell proliferation, cell cycle phases, brain development, differentiation, and neoplasia in various cell types [24]. In addition, GSL form “microdomains” or “rafts” within the cell membrane, which move within the fluid bilayer as platforms for the attachment of proteins during signal transduction and cell adhesion [24]. Thus, in this study, the inhibited glycosphingolipid biosynthesis metabolism seems to offer further proof that the communication or growth and differentiation of hepatocytes was potentially inhibited by the low-quality forage diet. The significance of the perturbation at a deeper level could not be ascertained by the results of the present study.

Inconsistent with the above 4 pathways, “Glycosaminoglycan biosynthesis - keratan sulfate” was highly activated in CS cows vs. MF cows (Fig. 2). Keratan sulfate (KS) is one of the glycosaminoglycans (GAG), occurring as keratan sulfate proteoglycans on the cell surface and in the extracellular matrix [25]. Pomin (2015) concluded that GAG displays anti-inflammatory functions by activating leukocyte rolling along the endothelial surface of inflamed sites and also regulating chemokine action on leukocyte guidance, migration and activation [26]. The study of Vailati-Riboni et al. (2016) in transition cows demonstrated that feeding at 125% of nutrient requirements activated hepatic GAG synthesis pathways in under-conditioned cows, while it inhibited it in optimally-conditioned cows [27]. Thus, it was suggested that overfeeding of fatter cows may decrease the synthesis of anti-inflammatory compounds and consequently induce some detrimental effects [27]. Taken together, previous and present data suggest activation of “Glycosaminoglycan biosynthesis - keratan sulfate” in response to feeding CS as an anti-inflammatory response. This idea was also validated by DAVID analysis where “complement activation” and “Complement and coagulation cascades” were significantly enriched with up-DEG (Fig. 3). However, the underlying mechanisms could not be ascertained from results of the present study.

The biosynthesis of KS is often markedly altered in response to metabolic, pathologic, or developmental changes in tissues [28]. Davies et al. (1999) suggested that the expression of keratan sulfate is down-regulated in migrating corneal endothelial cells, while abundance on the cell surface returns when cells cease migration [29]. Thus, this suggests that KS has an anti-migration character. However, the anti-adhesive properties of KS were previously reviewed by Caterson and Melrose (2018) and Funderburgh (2000) [28, 30]. Thus, the exact function of KS as it relates to cell-cell adhesion in hepatocytes is difficult to ascertain with the available data. In the present study, the paradoxical effect of CS vs. MF on cell adhesion was also highlighted by results of DAVID, where both “cell-cell adhesive” and “negative regulation of cell-matrix adhesion” were significantly enriched by the down- and up-regulated DEG, while “cell adhesive” was significantly enriched among the up-regulated DEG (Fig. 3).

### Low-quality forage inhibits amino acid metabolism

Metabolism of amino acids was overall inhibited by low-quality forage (Fig. 1). Among the top 20 impacted pathways, “Arginine biosynthesis”, “Selenoamino acid metabolism”, “beta-Alanine metabolism”, and “Tryptophan metabolism” were all inhibited (Fig. 2). Similar results were also revealed by ClueGO where “amide biosynthesis process” and “positive regulation of proteasomal protein catabolic” were significantly enriched by downregulated DEG (Fig. 4). Sun et al. (2015) studied metabolite profiles from four biofluids (rumen fluid, milk, serum, and urine) of cows fed different forage resources using metabolomics, with 55, 8, 28, and 31 significantly different metabolites identified in the rumen fluid, milk, serum, and urine, respectively [4]. These metabolites were involved in glycine, serine, and threonine metabolism; tyrosine metabolism; and phenylalanine metabolism [4]. Sun et al. (2016) in a subsequent urine metabolomics analysis demonstrated that Tyr metabolism and Phe, Tyr and Try biosynthesis pathways had the most variation when corn stover replaced alfalfa hay [31]. The study of Wang et al. (2018) showed that cows fed CS had lower absorbable Leu in the duodenum, which suggested this diet led to shortage of microbial Leu [8]. Sun et al. (2015) demonstrated that, under different quality forage resources, the concentrations of Phe and Tyr in rumen fluid exhibited lower fold-change values (0.54 and 1.19, respectively) than those in the serum (1.01 and 1.34, respectively), which implied that Phe and Tyr may be utilized more in the liver of cows fed high-quality forage than compared with low-quality forage [4]. Thus, we speculate that the inhibition of amino acid metabolism in CS vs. MF cows in this study was suggestive of an inhibited amino acid utilization in liver in the cows fed low-quality forage diet.

### Co-expression network analysis

In the co-expression network, degree represents the number of connections of a node in a network and betweenness centrality is the number of times that a path passes through the node, which represents the influence this node exerts over other nodes and their potential interactions in the network [32]. Thus, both degree and betweenness centrality are measures of the function of a node in network connectivity [33]. As shown in Fig. 5, the co-expression network revealed 7 genes (*FAM210A*, *SLC26A6*, *FBXW5*, *EIF6*, *ZSCAN10*, *FPGS*, *ARMCX2*) with higher degree and betweenness centrality (ranking in top 7, Additional file 5) than others, indicating a more critical role played by them in the network.

Among the 7 genes, *FAM210A* (a mitochondrial gene) which had the highest degree has a crucial role in regulating bone structure and function [34]. *SLC26A6* belongs to the solute carrier 26 family, and encodes a protein involved in transporting chloride, oxalate, sulfate and bicarbonate [35–39]. Thus, the inhibited expression of *SLC26A6* indicated a decreased transporting ability of chloride, oxalate, sulfate and bicarbonate in liver of CS cows (Fig. 5). FBXW5 is a the TSC2 binding receptor of CUL4 E3 ligase complex [40]. Hu et al. (2008) demonstrated that FBW5 (FBXW5) promotes ubiquitination of tumor suppressor TSC2 by DDB1-CUL4-ROC1 ligase, and depletion of FBW5 stabilizes TSC2 [41]. Ha et al. (2014) demonstrated that intracellular accumulation of TSC2 inhibits the activity of mTOR and increase autophagy [40]. Thus, in the present study, the downregulated *FBXW5* seems to imply an intracellular accumulation of TSC2 and consequently an increase in autophagy in liver of CS vs. MF cows. *EIF6* is eukaryotic translation initiation factor. Depletion of eIF6 (using specific siRNA-mediated knockdown) in Mz-ChA-2 and TFK-1 cell lines inhibit cell proliferation and induced apoptosis [42], while EIF6 over-expression increases the motility and invasiveness of cancer cells [43]. However, in this study, “positive regulation of apoptotic process” was significantly enriched by downregulated DEG implying that apoptosis was not induced by low-quality forage diet (Fig. 4). In addition, “protein folding” was significantly enriched by downregulated DEG (Fig. 4) and the pathway “Aminoacyl-tRNA biosynthesis” was highly inhibited by CS vs. MF. Thus, combined with the inhibited *EIF6*, the results of the present study suggested an inhibited protein synthesis in liver of cows fed low-quality forage.

*FPGS* is a gene encoding the folylpolyglutamate synthetase enzyme. This enzyme has a central role in establishing and maintaining both cytosolic and mitochondrial folylpolyglutamate concentrations and, thus, is essential for folate homeostasis and the survival of proliferating cells [44, 45]. Folate plays an essential role in nucleotide biosynthesis and biological methylation reactions as a methyl donor [46, 47]. Consistent with the downregulation of *FPGS* (Fig. 5), in this study, “Folate biosynthesis” was also inhibited by CS vs. MF (Fig. 2). Taken together, the results of the present study suggested a decrease in folate homeostasis in cows fed low-quality forage. Thus, the downregulation of “nucleotide-excision repair” may be a downstream cascade reaction due to decreased folate homeostasis (Fig. 3). In addition, the downregulated *ZSCAN10* and *ARMCX2* with high degree and betweenness centrality were also unraveled by co-expression network analysis (Fig. 5), but the significance of the perturbation was unclear.

Annotation information analysis for the genes within the co-expression network was performed using ClueGO and is shown in Fig. 6. A potential explanation for the perturbation of all these biological processes is beyond the scope of the present study. However, it is noteworthy that almost all these biological processes are energy-requiring except for “pyruvate metabolic process”, which is highly associated with energy metabolism. Furthermore, “pyruvate metabolic process” was also significantly enriched by the whole downregulated DEG in CS vs. MF (Fig. 4), indicating that energy metabolism in liver was inhibited by low-quality forage. Taken together, the inhibited energy metabolism unraveled by this study was suggestive of a central role in the whole metabolic perturbation in liver. The DMI of CS cows was indeed 4 kg/d lower than the MF cows, which may account at least in part for the decreased energy metabolism in liver in CS vs. MF.

## Conclusion

As in previous studies, feeding a low-quality forage reduces production performance, but also leads to marked alterations in the hepatic transcriptome. Among the unique biological pathways identified through bioinformatics analysis, glycan biosynthesis and metabolism and amino acid metabolism were highly inhibited when the low-quality forage diet was fed. Biological processes related to cell-cell adhesion, multicellular organism growth, and amino acid and protein metabolism also were downregulated. Co-expression network analysis indicated that the downregulated genes related to autophagy and translation played critical roles in the network. In addition, pyruvate metabolic process and other energy-requiring biological processes were enriched in the co-expression network. Collectively, results indicated that, compared to high-quality forage diet, low-quality forage could inhibit several basic cellular functions of the liver. Furthermore, the results of the present study provide an insight into the metabolic response in the liver to different-quality forage resources. As such, the data can help develop favorable strategies to improve the utilization of corn stover in China.

## Methods

### Experiment animals and management

The field experiment of this study was performed in Beijing ZhongDi Dairy Farm (Beijing, China) and the cows used in this study were all from this farm. Thirty-two healthy lactating Holstein cows (body weight, 550 ± 10 kg; days in milk, 55 ± 15; daily milk yield, 31 ± 2.30 kg, primiparous) were selected and divided into two groups based on average daily milk yield, body weight and days in milk. The two groups were randomly assigned to two diets: (i) mixed forage diet (MF), and (ii) corn stover diet (CS). Each treatment included 16 cows (*n* = 16). The two diets were formulated to meet their nutrient requirement (net energy lactation) according to NRC (2001) [3]. The ingredients and the chemical composition of the two experimental diets are shown in Table 2 and Table 3. Forage-to-concentrate ratio (F:C) of the two diets were all 64:36. The diets were mixed daily and fed ad libitum as total mixed ration. The diet was supplied thrice per day at 07:00, 14:00, and 20:00 h in an equal amount that allowed for 5–10% orts. Cows were milked thrice daily at 07:00, 14:00, and 20:00 h and had ad libitum access to water. The total duration of the experiment lasted for 14 weeks, including an acclimatization period of two weeks. All the two group cows were fed MF diet in acclimatization period and randomly allocated to MF or CS in trail period.

**Table 2:** Ingredients composition of experimental diets

Ingredient (% of DM)	MF	CS
Alfalfa hay	17.30	–
Corn silage	18.77	–
Corn straw	–	36.07
Soybean meal	11.29	11.29
Rapeseed meal	4.19	4.19
Cottonseed meal	2.13	2.13
Extruded soybeans	2.06	2.06
Beet pulp	4.16	4.16
Cottonseed fuzzy	10.44	10.44
Corn	25.55	25.56
EB100^a^	1.14	1.14
XP^b^	0.33	0.33
Limestone	0.74	0.74
Salt	0.46	0.46
Premix^c^	0.53	0.53
Total	100	100

^a^Mainly saturated free fatty acid fat supplement.

^b^Yeast products.

^c^Containing (per kilogram dry matter of premix): vitamin A 250,000 IU, vitamin D 65,000 IU, vitamin E 2100 mg, ferrum 400 mg, copper 540 mg, zinc 2100 mg, manganese 560 mg, iodine 35 mg; cobalt 68 mg

**Table 3:** Chemical composition of experimental diets

Items (% of DM)	MF	CS
DM	93.1	92.9
CP	18.1	16.1
NDF	35.9	47.6
ADF	25.2	30.3
EE	5.6	4.7
Starch	31.1	30.1
Ca	0.75	0.67
P	0.42	0.36
NEL, Mcal/kg of DM^a^	1.67	1.56

^a^calculated according to NRC (2001)

### Sample collection

Dry matter intake of each cow was recorded using the RIC system (Hokofarm Group, Netherlands). The offered total mixed rations were sampled twice per week, and the samples were pooled for each week and analyzed for DM, CP, NDF, ADF, EE, Starch, Ca, and P content as previously described [48]. Five cows were randomly selected from each treatment for liver tissue collection. The biopsy was conducted at the end of the 14 weeks as described by Gao et al. (2019) and Bu et al. (2017) [49, 50]. Tissue samples were washed with PBS buffer prepared using RNAase-free water, and then stored in liquid nitrogen immediately until RNA extraction. Health was monitored post-surgery by recording rectal temperature, milk yield, and feed intake daily for 7 days. Surgical clips were removed 7 days post-biopsy and the cows were placed back to their original barns in the farm to continue rear.

### RNA extraction and sequencing

Total RNA was extracted with TRIzol reagent (Life technologies, US, Cat#74106) according to the manufacturer’s protocol. Integrity and concentration of total RNA were then assessed using a 2100 Bioanalyzer (Agilent Technologies, US) with the RNA 6000 Nano Kit (Agilent Technologies, US). Complementary DNA (cDNA) was synthesised and used to construct a library with the NEBNext Ultra RNA Library Prep Kit (NEB, E7530). The libraries were sequenced on the Illumina HiSeq2000 platform via 2 × 50-bp paired-end sequencing at BGI Tech Solutions Co., Ltd. (Shenzhen, China). The RNA-Seq, libraries were sequenced at BGI Tech Solutions Co., Ltd. (Shenzhen, China).

### Quality analysis, mapping, and Transcriptome assembly

The reads containing adapter, poly-N and low-quality reads in the raw data were removed to obtain the clean reads. All the downstream analyses were based on the clean data with high quality. Reference bovine genome and gene model annotation files were downloaded from (ftp://ftp.ensembl.org/pub/release-89/fasta/bos_taurus/dna/) and (ftp://ftp.ensembl.org/pub/release-89/gtf/bos_taurus) respectively. Index of the reference genome was built using Bowtie2 v2.2.8. HISAT2 (v2.0.4) was used to align paired-end clean reads to the reference genome [51]. HISAT2 was run with ‘--rna-strandness RF’, other parameters were set as default. The StringTie (v1.3.1) was used to assemble the mapped reads of each sample in a reference-based approach [52].

### Statistical analysis

Data analysis of milk performance and milk composition was performed using the GLM model in SAS (8.2; SAS Institute Inc., Cary, NC), with treatment used as fixed effect, cow used as random effect, and day used as repeated effect.

#### Analysis of differently expressed genes (DEG)

The mapped reads count table of each gene and each sample was used for the analysis of DEG with DESeq2 package (1.26.0) in R (3.6.1) as the standard workflow instructed [53]. The complete dataset of DEG was in Additional file 1.

#### GO and KEGG enrichment analysis

Dynamic Impact Approach (**DIA**) was used to perform the analysis of Kyoto Encyclopedia of Genes and Genomes (KEGG) pathways [54]. The dataset including Entrez Gene ID, FDR (adjusted *P*-value, Padj), expression ratio, and *P*-value was uploaded and Entrez Gene ID were used as background. Adjusted *P*-value < 0.05 were used as cut-off. The output of DIA was exported in Additional file 2.

The enrichment analysis of various database including KEGG pathways, Gene Ontology Biological process (GO_BP), Cellular components (GO_CC) and Molecular function (GO_MF) was run by Database for Annotation, Visualization and Integrated Discovery (DAVID) v6.7 [55]. For this analysis, all the annotated transcripts that were detected (Entrez Gene ID) were used as background and three datasets were analyzed: 1) up-regulated differently expressed genes (DEG, log_2_ fold change > 0, Padj < 0.05) by CS vs MF; 2) down-regulated DEG (log_2_ fold change < 0, Padj < 0.05) by CS vs MF; and 3) both up- and down-regulated DEG (Padj < 0.05) of CS vs MF. Results were downloaded using both Functional Annotation Chart and Functional Annotation Clustering (Additional file 3). The enrichment analysis of Gene Ontology Biological process was also conducted and visualized using ClueGO (2.5.5) a plug-in of Cytoscape (3.7.2) [56].

#### Co-expression network construction and functional annotation

Read counts of each gene and each sample were normalized by median normalization using the EBSeq (3.10) R package. The correlation and correlation significance of every pair DEG (Padj < 0.01) was calculated using logarithmic matrix of read counts with the R package “psych” (1.8.12). Pearson was chosen as the correlation method. Then, the table containing the statistically significant correlations across the whole data set for every pair of DEGs was generated (Additional file 4). R package “igraph” (1.2.4.1) was used to calculate network statistics (degree and betweenness centrality for each node) as described by Contreras-Lopez et al. (2018) [33]. The dataset is available in Additional file 5. The dataset including correlation of the DEGs, the degree and betweenness centrality of the nodes, and the log2 fold change of the DEGs were uploaded to Cytoscape. The gene symbols were set as the node’s identifiers. The correlation, correlation significance, degree and betweenness, and log2 fold change were mapped to the edge color, edge with, node size, and node fill color respectively. The functional annotation analysis of the network was performed using the ClueGO application. The network and functional annotation results of the network were shown in Fig. 5 and Fig. 6.

## Supplementary Information

**Additional file 1.** Dataset of differently expressed genes (DEG) of CS vs. MF.

**Additional file 2.** Output of Dynamic Impact Approach (DIA) analysis of the DEG.

**Additional file 3.** Output of Database for Annotation, Visualization and Integrated Discovery (DAVID) analysis of the DEG.

**Additional file 4.** The table containing the statistically significant correlations across the whole data set for every pair of DEGs.

**Additional file 5.** Co-expression network statistics including correlation of the DEGs, the degree and betweenness centrality of the nodes.

## Data Availability

The datasets (additional files) supporting the conclusions of this article are available in the figshare, the hyperlink to datasets is 10.6084/m9.figshare.12082941.v2. All sequencing data (fastq files) generated in the present study are available in the NCBI Sequence Read Archive (SRA) (https://www.ncbi.nlm.nih.gov/sra/) with accession number SRP256053.

## Abbreviations

ADF: Acid detergent fiber
ARMCX2: Armadillo repeat containing X-linked 2
CUL4: Cullin 4
CP: Crude protein
CS: Corn stover
DAVID: Database for Annotation, Visualization and Integrated Discovery
DDB1: Damage specific DNA binding protein 1
DEG: Differently expressed genes
DIA: Dynamic Impact Approach
DM: Dry matter
DMI: Dry matter intake
EE: Ether Extract
EIF6: Eukaryotic translation initiation factor 6
FAM210A: Family with sequence similarity 210 member A
FBXW5: F-box and WD repeat domain containing 5
FDR: False discovery rates
FPGS: Folylpolyglutamate synthase
GAG: Glycosaminoglycans
GO_BP: Gene Ontology biological process
GO_CC: Gene Ontology cellular components
GO_MF: Gene Ontology molecular function
GSLs: Glycosphingolipids
KEGG: Kyoto Encyclopedia of Genes and Genomes
KS: Keratan sulfate
MCP: Microbial protein
MF: Mixed forage
Mz-ChA-2: Human gallbladder cancer
NDF: Neutral detergent fiber
NFC: Non-fibrous carbohydrates
RDP: Rumen degradable protein
ROC1: Rotamase CYP 1
RUP: Rumen undegradable proteins
SLC26A6: Solute carrier family 26 member 6
TFK1: Human extrahepatic bile duct carcinoma cell line
TSC2: TSC complex subunit 2
ZSCAN10: Zinc finger and SCAN domain containing 10
